# 3D DC geoelectric resistivity modeling with the singularity removal technique using finite element method based on orthosceme element

**DOI:** 10.1016/j.heliyon.2025.e42759

**Published:** 2025-02-18

**Authors:** Supriyadi, I.G.P.F. Soerya Djaja, Elfitra Desifatma, Harry Mahardika, Wahyu Srigutomo

**Affiliations:** Physics of Earth and Complex Systems, Physics Department, Faculty of Mathematics and Natural Sciences, Institut Teknologi Bandung, Jl. Ganesa 10, Bandung, 40132, Indonesia

**Keywords:** 3D DC geoelectric resistivity, Singularity removal, Orthosceme, Preconditioned conjugate gradients (PCG), Apparent resistivity

## Abstract

As a commonly used method for near-surface surveys, there is a demand for lightweight, fast, and accurate 3D DC geoelectric resistivity modeling that can be execute on portable computers. This study employed a singularity removal technique, solving the primary potential equation analytically and the secondary potential equation numerically using the finite element method. The modeling domain was discretized using orthosceme elements derived from dividing a hexahedron into six elements. The modeling domain (area of interest [AOI] + padding) covered [-300, 300] m × [-250, 250] m × [0, 250] m, divided into 59 × 39 × 20 nodes. The global matrix equation of the secondary potential is solved using the preconditioned conjugate gradients (PCG) with incomplete Cholesky factorization (ICF) as a preconditioner, making it 6.7 times faster than the direct solver. The combination of the singularity removal, orthosceme discretization, and the PCG solver enabled lightweight, fast, and accurate 3D resistivity modeling on a portable computer. Benchmarking this modeling against a layered Earth model showed that the Wenner and Schlumberger arrays achieved apparent resistivity with a relative error of mostly <5 %. Applying this modeling to the vertical contact and buried block 3D anomaly Earth models demonstrated that the apparent resistivity profiles from Wenner, Schlumberger, and dipole-dipole arrays effectively captured resistivity changes at the anomaly boundaries, aligning with previous studies.

## 1 Introduction

The Direct Current (DC) geoelectric resistivity method is a geophysical technique frequently employed in near-surface investigations. The basic principle of the DC geoelectric resistivity method involves injecting a DC electric current through two current electrodes into the Earth's medium and measuring the voltage response produced by two potential electrodes [1]. When an electric current is injected into the Earth's medium, potentials are generated and dispersed according to the electrical properties of the medium and the spatial function described by the Poisson equation. For *N* points current source at a location *x*_*i*_ with a current *I*_*i*_, the Poisson equation is as follows:

(1) $$-\nabla ∙\left(\sigma \nabla V\right)=\sum_{i=1}^{N}I_{i}\delta_{x_{i}},\sum_{i=1}^{2}I_{i}\delta_{x_{i}}=I\;\left\{\delta \left(\vec{r}-{\vec{r}}_{s+}\right)-\delta \left(\vec{r}-{\vec{r}}_{s-}\right)\right\}$$

where *V* is the electric potential, *I*_*i*_ is the *i*-th current source, $\delta_{x_{i}}$ is *i*-th spatial position of the current source. A pair of current electrodes (dipole, *N* = 2) with opposing polarity, +*I* (source) and −*I* (sink), are commonly used to perform the DC geoelectric resistivity method. The electric potential is caused by the electric field induced by the dipole, which is expressed as two Dirac delta functions centered at the positive (${\vec{r}}_{s+}$) and negative (${\vec{r}}_{s-}$) source points [[2], [3], [4]].

Poisson equation (Eq. (1)) for the 3D DC geoelectric resistivity method has a differential operator concerning potential on the left side and a source term expressed as a Dirac delta function on the right side. The Dirac delta function used in the current source term leads to a singularity issue in the solution, which is a critical challenge in computing the DC resistivity response. One alternative to mitigate this problem is mesh refinement around the current sources, which increases the computational load [2,5,6]. This poses a significant challenge when computations are performed on portable computers. Another continuously developed alternative is the singularity removal technique introduced by Lowry et al. [7] and further refined by Zhao and Yedlin [8]. To address the singularity problem, the potential is split into two parts: a primary potential resulting from the current source and a secondary potential resulting from the inhomogeneous conductivity of the Earth's medium. The singularity removal technique was initially applied in the finite element method by Li and Spitzer [9], leading to significantly enhanced modeling accuracy. Development and application of the singularity removal technique continue in 3D DC geoelectric resistivity modeling using finite element methods, as demonstrated by Wu [10], Rücker et al. [11], Blome et al. [12], Wang et al. [13], Penz et al. [14], Schaa et al. [15], Song et al. [16], and Ren et al. [17]. Most researchers have successfully implemented the singularity removal technique on unstructured tetrahedron elements using the mesh generator TetGen, such as Rücker et al. [11], Blome et al. [12], Wang et al. [13], and Ren et al. [17]. However, some researchers, such as Wu [10] and Song et al. [16], still prefer structured tetrahedron elements because they are easier to formulate without a mesh generator and require less computing load. In this study, structured tetrahedron elements are obtained by dividing the cube into six tetrahedrons called orthosceme. Despite the singularity arising from the current source and the element formulation issues, the 3D DC geoelectric resistivity modeling using the finite element method also faces challenges related to the computational load of solving large-scale sparse linear equations. Most previous researchers have used iterative solvers, such as Wu [10], Rücker et al. [11], Song et al. [16], and Yang et al. [18], while some still use direct solvers, such as Blome et al. [12] and Acka and Gölebatmaz [19]. Although lightweight computers have made direct solver relatively fast, the necessity for repetitive forward modeling due to changes in the current source position and iterations in inversion promotes the use of iterative solvers, even with additional preconditioners. Some researchers, such as Xi et al. [20], Gould and Scott [21], and Gu et al. [22] specifically developed and tested preconditioners for iterative solvers. However, this study opts for ready-to-use iterative solvers and preconditioners for simplicity.

The combined use of the singularity removal technique, orthosceme-structured elements, and specific iterative solvers in 3D DC geoelectric resistivity modeling running on a lightweight portable computer/laptop is expected to maintain the speed and accuracy of the solution. The potential at any point on the Earth's surface is calculated through nodes interpolation with an area ratio scheme to ensure flexibility in electrode array selection. Modeling is performed on a layered Earth model as an accuracy test benchmark using Wenner and Schlumberger arrays. The performance of Wenner, Schlumberger, and dipole-dipole arrays is also analyzed on a vertical contact and a buried block 3D anomaly Earth model.

## 2 Singularity removal technique

The differential equation has a singular solution centered on the current source point because the right side of Eq. (1) is a Dirac delta function. Direct numerical approaches often give poor results near singularities, even for flat homogeneous Earth model. The percentage error of the numerical solution is relatively large near the current injection point, i.e., <1 m [23]. Lowry et al. [7] proposed a singularity removal technique address this issue. This technique leverages the linearity in Eq. (1) by splitting the potential *V* into two components:

(2) $$V\left(\vec{r}\right)=V_{p}\left(\vec{r}\right)+V_{s}\left(\vec{r}\right)$$

where *V*_*p*_ is the singular part and *V*_*s*_ is the standard potential or non-singular part. Zhao and Yedlin [8] provide a better definition of these potentials. *V*_*p*_ is primary potential caused by the current source in a homogeneous half-space with conductivity *σ*_*p*_ and *V*_*s*_ is secondary potential caused by the inhomogeneity of conductivity (secondary conductivity, *σ*_*s*_ = *σ− σ*_*p*_).

The primary potential *V*_*p*_ is derived from based on Eq. (1) and is formulated as:

(3) $$-\nabla ∙\left(\sigma_{p}\nabla V_{p}\right)=I\;\left\{\delta \left(\vec{r}-{\vec{r}}_{s+}\right)-\delta \left(\vec{r}-{\vec{r}}_{s-}\right)\right\}$$

The calculation of primary potential *V*_*p*_ can be performed analytically for the flat Earth model, which represents the response for a homogeneous half-space with conductivity *σ*_*p*_. Primary conductivity *σ*_*p*_ is defined as the conductivity of the medium at the singular point [8]. The analytic solution of Eq. (3) is given by:

(4) $$V_{p}=\frac{I}{2\pi \sigma_{p}}\left(\frac{1}{r_{+}}-\frac{1}{r_{-}}\right)$$

where *r*_*+*_ and *r*_*-*_ are the distances between the observation point and the current source points. The current dipole + *I* is located at *r*_*s+*_(*x*_*s+*_*, y*_*s+*_*,*0) and −*I* is located at *r*_*s−*_(*x*_*s−*_*, y*_*s−*_*,*0). For non-flat Earth models, the primary potential *V*_*p*_ is commonly calculated numerically [11]. The equation for the secondary potential *V*_*s*_ is obtained by substituting Eq. (2) into Eq. (1) and then subtracting it from Eq. (3) to obtain Eq. (5). The equation is as follows:

(5) $$\nabla ∙\left(\sigma \nabla V_{s}\right)=\nabla ∙\left(\left(\sigma_{p}-\sigma \right)\nabla V_{p}\right)$$

**Fig. 1:**
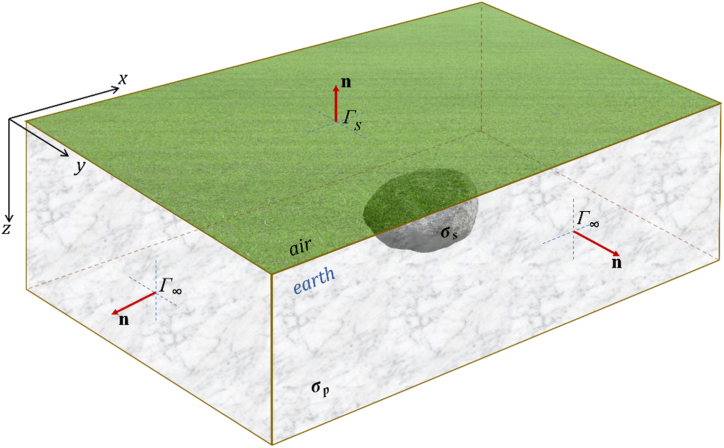
3D DC geoelectric resistivity modeling domain and boundary conditions are Neumann in the plane of the air-Earth surface (*Г*_s_) and Dirichlet in side and bottom planes (*Г*_∞_).

The differential equations for primary potential *V*_*p*_ (Eq. (3)) and secondary potential *V*_*s*_ (Eq. (5)) can be solved numerically using finite element method by applying appropriate boundary conditions (Fig. 1). Since air is non-conductive, the electric flux n∙∇V is zero at the air-Earth surface boundary (*Г*_**s**_) as a Neumann boundary condition (Eq. (6)) [7,14,15,24]:

(6) $$\begin{array}{c} \sigma_{p}\;n∙\nabla V_{p}=0\\ \;\sigma \;n∙\nabla V_{s}=0 \end{array}\}\;at\;\Gamma_{S}$$

where ***n*** is normal of the air-Earth surface boundary (*Г*_**s**_). For other boundary conditions *Г*_**∞**_ (at side *x* = ±∞, *y* = ±∞, and bottom *z* = +∞), mixed boundary conditions cannot be applied because the expression of the non-singular potential field along these boundaries is unknown [12]. Providing the modeling domain is sufficiently large by adding padding, Dirichlet boundary conditions can be applied, i.e., the potential approaches zero at the side and bottom boundaries *Г*_**∞**_ that are far enough from the source (Eq. (7)) [14,15,24]:

(7) $$V=0\;;{\;V}_{s}=-V_{p}\;at\;\Gamma_{\infty }$$

## 3 Discretization and interpolation function in finite element method

**Fig. 2:**
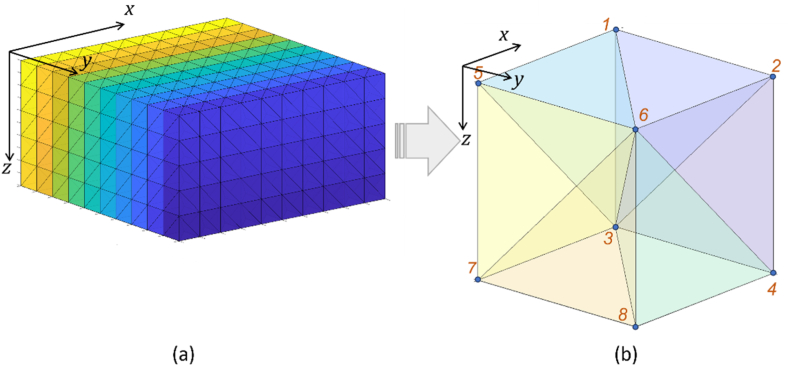
(a) Structured orthosceme grid derived by splitting each hexahedron into six tetrahedrons, (b) The descriptive codes of this orthosceme are (1 6 5 3), (3 6 5 7), (3 6 7 8), (1 2 6 3), (2 4 6 3), and (6 3 4 8).

In finite element implementation, the modeling domain (Fig. 1) is discretized, which involves dividing the 3D modeling domain into smaller parts, namely 3D elements. There are several alternative choices of 3D elements that are commonly used, including hexahedron, tetrahedron, and triangular prism [25]. This study uses structured elements that are self-constructed without a mesh generator to facilitate understanding of the element setup rules, which will be further utilized in the inversion scheme. Structured tetrahedrons can be obtained by splitting each hexahedron grid (cube) into five or six tetrahedrons. A type of six tetrahedrons from one hexahedron (Fig. 2) following the element construction scheme by Wang et al. [13] is employed in this study. Each tetrahedron created has three edges mutually perpendicular to each other, connecting all its vertices, thus belonging to the orthosceme type. Since it is acquired from a cube, three mutually perpendicular edges have a length of one unit, i.e., cube edge, two edges with a length of √2, i.e., plane diagonal, and one edge with a length of √3, i.e., space diagonal. A tetrahedron is categorized as an orthosceme if it has three mutually perpendicular edges without a shared common vertex [26,27]. Hereafter, the term tetrahedron refers to the orthosceme type tetrahedron.

An interpolation function is used to determine the unknown quantity (i.e., potential *v*) for each orthosceme element in a 3D DC geoelectric resistivity modeling using the finite element method. The interpolation function must ensure that potential *v* is continuous in the domain and can be derived at least once. The potential value is obtained by interpolating the potential values at each node of each element, as shown in Eq. (8):

(8) $$v=\sum_{j}v_{j}^{e}N_{j}$$

where *v* is the potential value inside an element, $v_{j}^{e}$ is the potential values at node in an element, and *N*_*j*_ is the interpolation function of each node. In the construction grid scheme of elements as six orthosceme from a hexahedron (Fig. 2b), each element consists of four nodes constituting the vertex points of a hexahedron. The interpolation function belongs to linear interpolation because it only involves two nodes for each interpolation function. This choice of orthosceme element and linear interpolation function provides a fast discretization process.

**Fig. 3:**
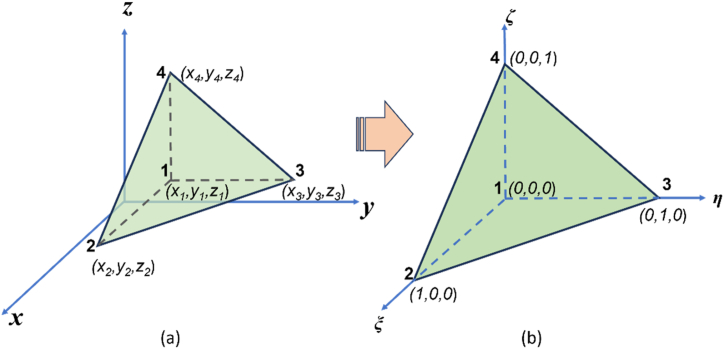
(a) Orthosceme on *x-y-z* coordinates (left) mapped to (b) Master orthosceme on *ξ-η-ζ* coordinates (right) (adopted from Srigutomo et al. [28]).

The modeling domain is expressed in *x-y-z* coordinates, where the position of each orthosceme element corresponds to a specific *x-y-z* coordinate. To simplify the handling of each orthosceme, the elements in local coordinates *x-y-z* are mapped into natural coordinates *ξ-η-ζ* and are called master elements (Fig. 3aandb). The interpolation function *N*_*j*_ in natural coordinates for each node is shown in Eq. (9):

(9) $$N_{1}\left(\xi ,\eta ,\zeta \right)=1-\xi -\eta -\zeta \;N_{2}\left(\xi ,\eta ,\zeta \right)=\xi \;N_{3}\left(\xi ,\eta ,\zeta \right)=\eta \;N_{4}\left(\xi ,\eta ,\zeta \right)=\zeta$$

The coordinate transformation from *x-y-z* to *ξ-η-ζ* can be obtained from Eqs. (8), (9). This coordinate transformation is further used to calculate the derivative of the interpolation function (Eq. (10)).

(10) $$\left(\begin{array}{c} \frac{\partial N}{\partial x}\\ \frac{\partial N}{\partial y}\\ \frac{\partial N}{\partial z} \end{array}\right)={\overset{⃡}{J}}^{-1}\left(\begin{array}{c} \frac{\partial N}{\partial \xi }\\ \frac{\partial N}{\partial \eta }\\ \frac{\partial N}{\partial \zeta } \end{array}\right),\overset{⃡}{J}=\left(\begin{array}{ccc} {\bar{x}}_{21} & {\bar{y}}_{21} & {\bar{z}}_{21}\\ {\bar{x}}_{31} & {\bar{y}}_{31} & {\bar{z}}_{31}\\ {\bar{x}}_{41} & {\bar{y}}_{41} & {\bar{z}}_{41} \end{array}\right)$$

The right side of Eq. (10) is summarized in Table 1**.**

**Table 1:** The derivative of each interpolation function to each natural coordinate.

	$N_{1}$	$N_{2}$	$N_{3}$	$N_{4}$
$\frac{\partial N_{j}}{\partial \xi }$	−1	1	0	0
$\frac{\partial N_{j}}{\partial \eta }$	−1	0	1	0
$\frac{\partial N_{j}}{\partial \zeta }$	−1	0	0	1

## 4 Finite element method formulation for secondary potential $V_{s}$

Using the singularity removal technique, the 3D DC geoelectric resistivity problem is solved through the primary and secondary potentials (Eqs. (3), (5)). Some researchers have used numerical methods to solve the primary potential equation [11,15], while others suggest that the flat Earth model can be solved analytically [9,10,13,14]. An analytical solution for the primary potential promises to improve the speed and accuracy of 3D DC geoelectric resistivity modeling, with only secondary potential equation being solved numerically using the finite element method with the Galerkin approach.

Using the weighted residual method with Galerkin's approach [25,28], the differential equation is generally expressed as Eq. (11).

(11) $$\frac{\partial }{\partial x}\left(\beta_{x}\frac{\partial v}{\partial x}\right)+\frac{\partial }{\partial y}\left(\beta_{y}\frac{\partial v}{\partial y}\right)+\frac{\partial }{\partial z}\left(\beta_{z}\frac{\partial v}{\partial z}\right)=g$$

For secondary potential (Eq. (5)), parameters in Eq. (11) are defined as unknown variables *v* = *V*_*s*_, constant *β*_*x*_ = *β*_*y*_ = *β*_*z*_ = *σ*, and $g=\frac{\partial }{\partial x}\left(\left(\sigma_{p}-\sigma \right)\frac{\partial V_{p}}{\partial x}\right)+\frac{\partial }{\partial y}\left(\left(\sigma_{p}-\sigma \right)\frac{\partial V_{p}}{\partial y}\right)+\frac{\partial }{\partial z}\left(\left(\sigma_{p}-\sigma \right)\frac{\partial V_{p}}{\partial z}\right)$ with $\sigma_{p}-\sigma =\gamma$ and *u* = *V*_*p*_. The primary potential *V*_*p*_ is calculated analytically using Eq. (4) at each node. Using the finite element formulation with the Galerkin approach, where the weight function *w* = *N*_*i*_, the secondary potential equation for an element is obtained as in Eq. (12).

(12) $$\left[\begin{array}{c} L_{11}^{e}\\ L_{21}^{e}\\ \vdots \\ L_{n1}^{e} \end{array}\begin{array}{c} L_{12}^{e}\\ L_{22}^{e}\\ \vdots \\ L_{n2}^{e} \end{array}\begin{array}{c} \cdots \\ \cdots \\ \ddots \\ \cdots \end{array}\begin{array}{c} L_{1n}^{e}\\ L_{2n}^{e}\\ \vdots \\ L_{nn}^{e} \end{array}\right]\left\{\begin{array}{c} v_{1}^{e}\\ v_{2}^{e}\\ \vdots \\ v_{n}^{e} \end{array}\right\}=\left[\begin{array}{c} M_{11}^{e}\\ M_{21}^{e}\\ \vdots \\ M_{n1}^{e} \end{array}\begin{array}{c} M_{12}^{e}\\ M_{22}^{e}\\ \vdots \\ M_{n2}^{e} \end{array}\begin{array}{c} \cdots \\ \cdots \\ \ddots \\ \cdots \end{array}\begin{array}{c} M_{1n}^{e}\\ M_{2n}^{e}\\ \vdots \\ M_{nn}^{e} \end{array}\right]\left\{\begin{array}{c} u_{1}^{e}\\ u_{2}^{e}\\ \vdots \\ u_{n}^{e} \end{array}\right\}+\left\{\begin{array}{c} q_{1}^{e}\\ q_{2}^{e}\\ \vdots \\ q_{n}^{e} \end{array}\right\}+\left\{\begin{array}{c} r_{1}^{e}\\ r_{2}^{e}\\ \vdots \\ r_{n}^{e} \end{array}\right\}$$

The calculation of $L_{ij}^{e}$ elements are obtained by integrating them in natural coordinates with the assistance of Jacobian transformation. The volume integral of the orthosceme element is 1/6 so $L_{ij}^{e}$ elements become as in Eq. (13),

(13) $$L_{ij}^{e}=\frac{\left|J\right|}{6}\left[\beta_{x}\frac{\partial N_{i}}{\partial x}\frac{\partial N_{j}}{\partial x}+\beta_{y}\frac{\partial N_{i}}{\partial y}\frac{\partial N_{j}}{\partial y}+\beta_{z}\frac{\partial N_{i}}{\partial z}\frac{\partial N_{j}}{\partial z}\right]$$

where |J| is obtained from Eq. (10) and is a constant, while the derivative of *N* is obtained from Eq. (10) and Table 1. The $M_{ij}^{e}$ elements are obtained by a similar procedure and the results are

written as in Eq. (14).

(14) $$M_{ij}^{e}=\frac{\left|J\right|}{6}\left[\gamma_{x}\frac{\partial N_{i}}{\partial x}\frac{\partial N_{j}}{\partial x}+\gamma_{y}\frac{\partial N_{i}}{\partial y}\frac{\partial N_{j}}{\partial y}+\gamma_{z}\frac{\partial N_{i}}{\partial z}\frac{\partial N_{j}}{\partial z}\right]$$

Vector elements $q_{i}^{e}$ and $r_{i}^{e}$ in Eq. (12) are evaluated only at the boundary of the modeling domain with Neumann boundary conditions (Eq. (6)), where σn∙∇v=0 and $\sigma_{p}\;n∙\nabla u=0$ on air-Earth's surface boundary (*Г*_**s**_, Fig. 1), all elements of $q_{i}^{e}$ dan $r_{i}^{e}$ are zero.

The global matrix system equation is obtained by summing the matrix equations of each element (Eq. (12)) for all elements in the modeling domain. The summations are done sequentially, starting by summing the equation of element 1 with the equation of element 2, the result is summed again with the equation of element 3, and so on until element 6, referring to the connectivity matrix, i.e., descriptive codes in Fig. 2b. The summations are also performed between cubic hexahedrons for the entire modeling domain (Fig. 2a) so that the global matrix equation of the secondary potential is obtained as in Eq. (15),

(15) $$\vec{L}\vec{v}=\vec{d}$$

with $\vec{L}=\sum_{e}{\vec{L}}^{\left(e\right)}$ and $\vec{d}=\sum_{e}{\vec{d}}^{\left(e\right)}=\vec{M}\vec{u}$. The implementation of the Dirichlet boundary condition (*Г*_**∞**_, Fig. 1) involves the setting of a value $v=V_{s}=-V_{p}$ (Eq. (7)) at side and bottom boundary nodes, using Özgün and Kuzuoğlu's algorithm [25]. This reshapes the global matrix into Eq. (16).

(16) $${\vec{L}}_{ok}\vec{v}={\vec{d}}_{ok}$$

The global matrix equation comprises a set of linear equations of secondary potential for all elements in the modeling domain, making it large, sparse, and real symmetric [5,18,23,[29], [30], [31]]. The solution of Eq. (16) can be obtained using an appropriate iterative solver. The iterative solver options for the real symmetric case include the conjugate gradient (CG) method, the minimal residual (MINRES) method, and the SYMMLQ method [32,33].

After calculating the primary potential $u=V_{p}$ analytically and the secondary potential $v=V_{s}$ numerically, the total potential is obtained by summing both using Eq. (2). The DC electric current injected into the Earth's medium with varying resistivity is physically described by the potential distribution. The influence of electric current injection and Earth's medium resistivity can be observed through the potential distribution pattern in the Earth's medium. For further use of these modeling results, only the potential distributions on the Earth's surface are utilized, as measured in DC geoelectric resistivity data collection in the field. These potential values are used to calculate apparent resistivity using specific electrode arrays such as Wenner, Schlumberger, dipole-dipole, etc.

## 5 Handling the numerical potential solution

**Fig. 4:**
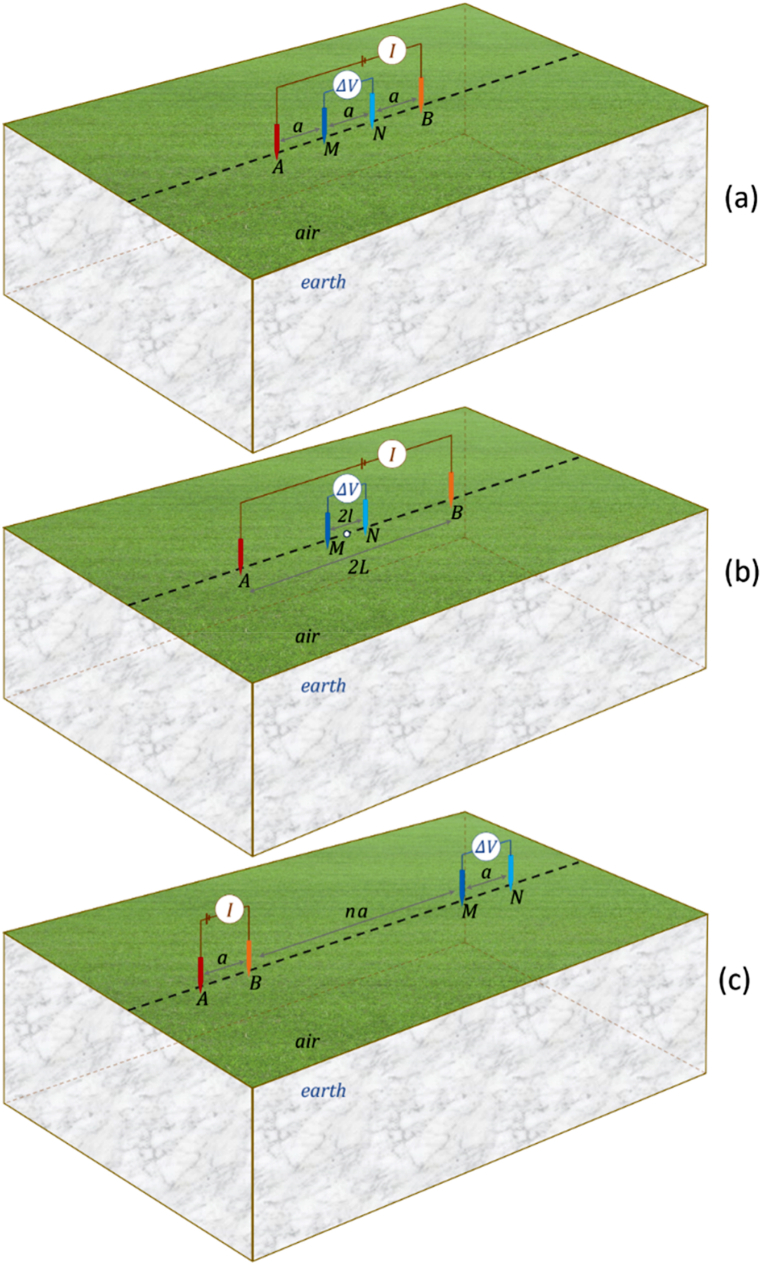
Electrode arrays used in 3D DC geoelectric resistivity modeling include (a) Wenner, (b) Schlumberger, and (c) dipole-dipole.

The geoelectric resistivity method is practically applied using specific electrode arrays. In this study, the 3D DC geoelectric resistivity modeling utilizes Wenner, Schlumberger, and dipole-dipole arrays (Fig. 4a–c). All of these arrays consist of two current and two potential electrodes. The position of current electrodes and potential electrodes on the Earth's surface is determined according to these array rules. Any position of the current electrodes can be easily accommodated because the primary potentials are calculated analytically. Numerical modeling of 3D DC geoelectric resistivity using the finite element method only produces a potential solution at a node. Therefore, mathematical handling is necessary to obtain the solution value at any point based on specific electrode array rules.

**Fig. 5:**
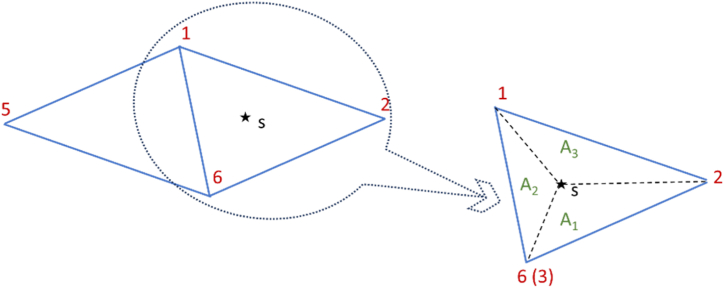
Numbering of elements occupied by a potential electrode and calculating the interpolation function using the area ratio scheme to handle potential value. The left image shows the top of elements in a one hexahedral block on the Earth's surface where electrodes are placed.

Based on the descriptive codes for orthosceme tetrahedron in Fig. 2b, the position of electrodes on Earth's surface (*z* = 0) is only feasible on the top surface of element 1 with nodes (1 6 5 3) and element 4 with nodes (1 2 6 3) (Fig. 5−left). Suppose point S is the electrode point. Using the concept of cross product between the vector of node 1 to node 6 and the vector of node 1 to point S, we can determine whether the electrode is located in element 1 or element 4 of a cube. If the cross product of the two vectors is less than zero, the electrode point lies in element 1. Otherwise, if the result is greater than or equal to zero, the electrode point lies in element 4. The numbering of elements 1 or 4 is further converted into a global element number for the arrangement of elements in the entire modeling domain.

The potential at a specific electrode can be obtained by interpolating the numerical values of potential at all nodes of the element where the electrode is located (as shown in Eq. (8)). The interpolation function is calculated using an area ratio scheme, as shown in Fig. 5−right [34,35]. The linear interpolation function can be expressed by area ratio, as in Eq. (17),

(17) $$N_{j}^{e}\left(x,y\right)=\frac{A_{j}^{e}}{A_{tot}^{e}},j=1,2,3$$

where $A_{j}^{e}=\left(\frac{1}{2}\right)\left(r-r_{j+1}^{e}\right)\;\left(r_{j-1}^{e}-r_{j+1}^{e}\right)∙\hat{z}$ and $A_{tot}^{e}=\sum A_{j}^{e}$. For two potential electrodes, i.e., M and N, potentials are written as in Eq. (18).

(18) $$V_{M}\left(x,y\right)=\sum_{i=1}^{3}V_{j}^{e}N_{j}^{e}\;V_{N}\left(x,y\right)=\sum_{i=1}^{3}V_{j}^{e^{\prime }}N_{j}^{e^{\prime }}$$

## 6 Results and discussion

### 6.1 Layered earth model as modeling benchmarking

Applying the singularity removal technique in solving 3D DC geoelectric resistivity problems for a homogeneous Earth model causes the right-hand side of Eq. (5) to be zero. As a result, there is no secondary potential solution, so the total potential solution is solely contributed by the primary potential, which is calculated analytically. The layered Earth model presents challenges in obtaining the analytical potential, so the modeling accuracy test is conducted using the apparent resistivity of the Wenner and Schlumberger arrays. The apparent resistivity of the Wenner array for a two-layer Earth model has been well-established analytically by Telford et al. [36]. The apparent resistivity of the Schlumberger array is analytically obtained by solving the Hankel integral [37], addressing both the two-layer and three-layer Earth models.

**Fig. 6:**
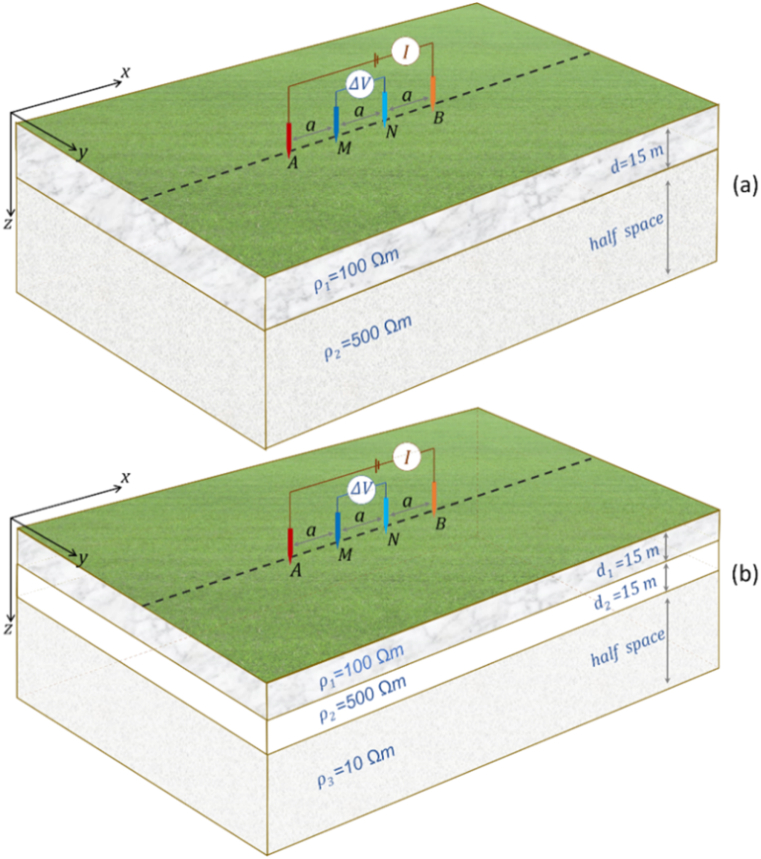
(a) Two-layer Earth model, and (b) three-layer Earth model, with Wenner as an example of the array used for modeling benchmarking. The Schlumberger array is also applied to both layered Earth models for modeling benchmarking.

**Fig. 7:**
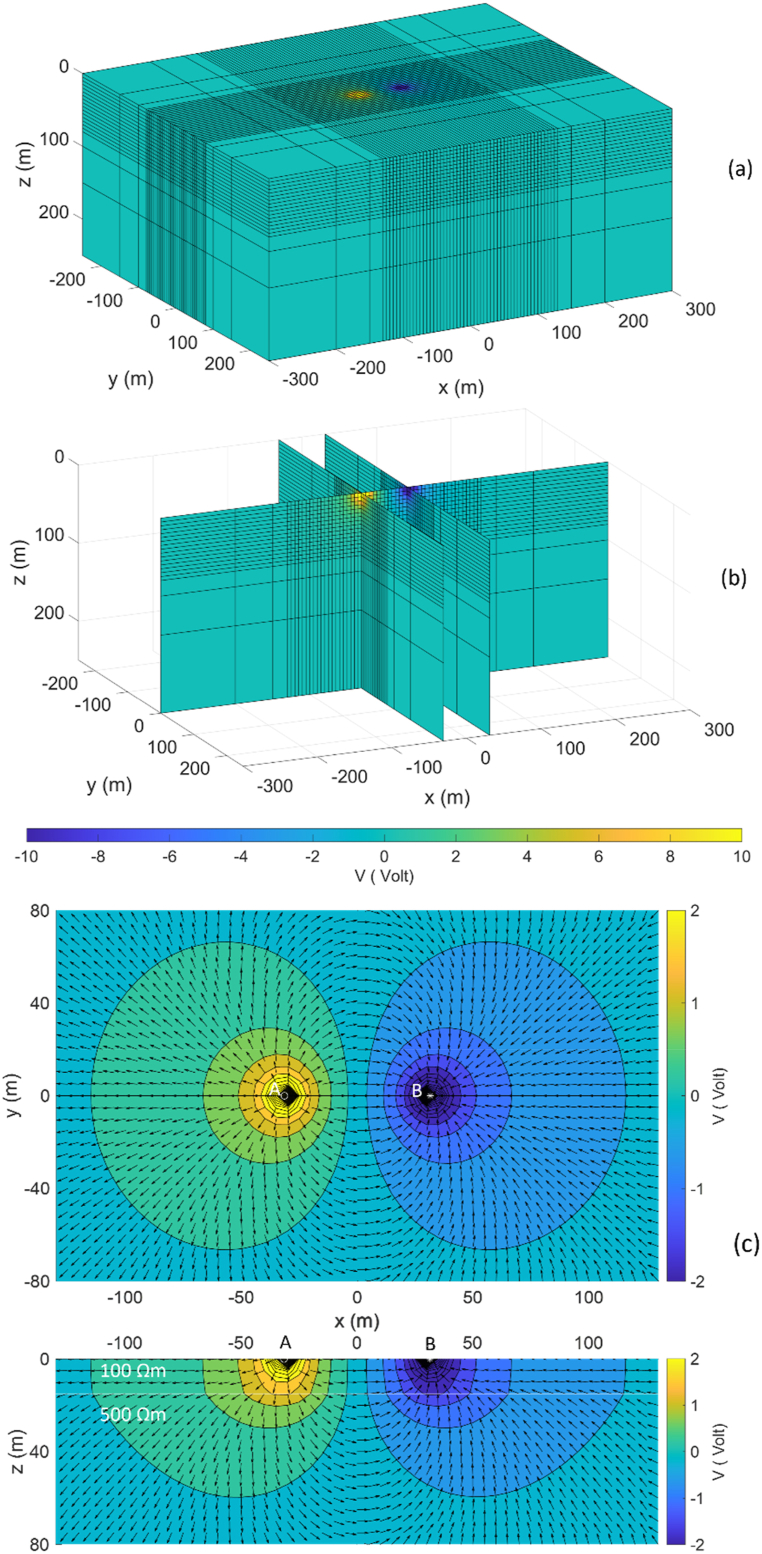
The 3D potential distribution for a two-layered Earth model using the Wenner array, with spacing *a* = 20 m and *I* = ± 1A (a) edge surfaces view, (b) intersections of current injection view, and (c) normalized current flow and potential distribution pattern on surface view and intersection view at *y* = 0 only on AOI.

The Wenner array is defined by the same distance of AM, MN, and NB electrodes, denoted as *a*. A DC electric current of ±1A is injected through current electrodes A and B, resulting in a potential response measured at potential electrodes M and N. The layered Earth models (Fig. 6aandb), vertical contact Earth model, and buried block 3D anomaly Earth model are realized in the modeling domain *x* = [−130, 130] m, *y* = [−80, 80] m, and *z* = [0, 80] m as the area of interest (AOI). The modeling domain is divided into equidistant structured hexahedron grids with a size of Δ*x* = Δ*y* = Δ*z* = 5 m, resulting in 52 × 32 × 16 blocks. For the orthosceme element, where a hexahedron is divided into six elements, there are 52 × 32 × 16 × 6 = 159,744 elements. This scheme of element construction does not increase the number of nodes, which remains 53 × 33 × 17 nodes. To ensure the applicability of the Dirichlet boundary condition, three padding nodes are added at gradually increasing distances at each border of the AOI except Earth's surface (Fig. 7aandb). The addition of this padding causes the number of nodes to become 59 × 39 × 20 nodes, and the modeling domain becomes *x* = [−300, 300] m, *y* = [−250, 250] m, and *z* = [0, 250] m. *These numerical modeling efforts for layered, vertical contact, and buried block 3D anomaly block Earth models run on portable computers/laptops* with specifications, i.e., AMD Ryzen 5 5600H processor (six cores, 3.3 GHz) and 16 GB RAM, resulting in a relatively low the computational load.

In the DC geoelectric resistivity method, each array electrode generally involves changing the spacing and position of the current electrodes. The vector ${\vec{d}}_{ok}$ (Eq. (16)) only handles information for a pair of current electrode positions. Therefore, it is necessary to compute the global matrix solution every time a pair of current electrode positions changes. The properties of the global matrix and the repetitive computations encourage the use of iterative solvers to ensure speed and accuracy. The selection of an appropriate iterative solver is based on the properties of Eq. (16). Some researchers claim that the global matrix in Eq. (16) is positive definite [5,18,23,29], in addition to the properties previously mentioned. Mathematical tests on the layered Earth model (Fig. 6a) with the current electrode spacing of the Wenner array *a* = 2 m and 59 × 39 × 20 nodes (AOI and padding) confirmed the global matrix properties, showing that it is large-scale, sparse, real symmetric, and positive definite. These properties also hold for other Earth models and electrode arrays. The best iterative solver for Eq. (16) among the three previously mentioned options is the CG method [14,[31], [32], [33],^38^]. The addition of padding in the modeling domain necessitates using preconditioners in the CG method to accelerate convergence and maintain accuracy [39]. Incomplete Cholesky factorization (ICF) is an appropriate preconditioner for symmetric positive definite [9,11,39,40]. A minor modification was made to the zero fill-in variant in the ICF to enhance convergence speed. To better illustrate the performance of the preconditioned conjugate gradient (PCG) solver compared to the direct solver, the runtime of the PCG is evaluated from the ICF generation to the completion of solving Eq. (16). For forward modeling of the two layered Earth model with the current electrode spacing of the Wenner array *a* = 2 m, the direct solver has an average runtime of 0.366394 s over twenty runs. In contrast, the PCG with ICF as a preconditioner, has an average runtime of 0.054628 s and converges at the 43rd iteration with a relative residual of 7.7 × 10^−7^. The PCG is 6.7 times faster than the direct solver. For a more comprehensive illustration of their performance across various global matrix's size, a detailed runtime comparison between the two solvers is provided in Appendix A. Due to the repeated computation to accommodate a particular electrode array, especially if used in the inversion scheme, the PCG with ICF significantly speeds up computation time while maintaining accuracy. The PCG with ICF is employed for all modeling in this study.

The 3D DC geoelectric resistivity modeling solution with the singularity removal technique for a two-layer Earth model as potential distribution is provided in 3D view, i.e., edge surfaces view (Fig. 7a) and intersections of the current injection view (Fig. 7b). *Based on* Fig. 7a and b*, the potential distribution pattern is characterized by the largest positive and negative potentials centered on the current electrodes, with the potential value decreases as the distance from as a function of spatial distance and conductivity of the medium. The effect of resistivity variation of each layer on potential distribution patterns is difficult to observe, even at layer boundaries.* The Wenner and Schlumberger arrays yield similar potential solutions but with different positions of the largest positive and negative potentials, as well as for the three-layer Earth model. The potential solution remains similar for the same electrode spacing on both arrays, so it suffices to visualize the solution using the Wenner array. Fig. 7c provides a better illustration because it focuses solely on the AOI area without involving padding, and the potential is expressed in a smaller limit range from −2 to 2 V to provide a clear visualization of the potential variation, except in the area near the current electrode due to rapid changes. Additionally, the relationship between normalized current flow and the resulting potential distribution pattern at each point can explain the influence of spatial distance and medium resistivity in the modeling domain. Based on the direction of current flow expressed by arrows, the current spreads in all directions from the positive current electrode and converges back at the negative current electrode through the Earth medium with a specific resistivity. The direction of current flow and equipotential line patterns experience significant changes and can be well observed at layer boundaries with different resistivities.

**Fig. 8:**
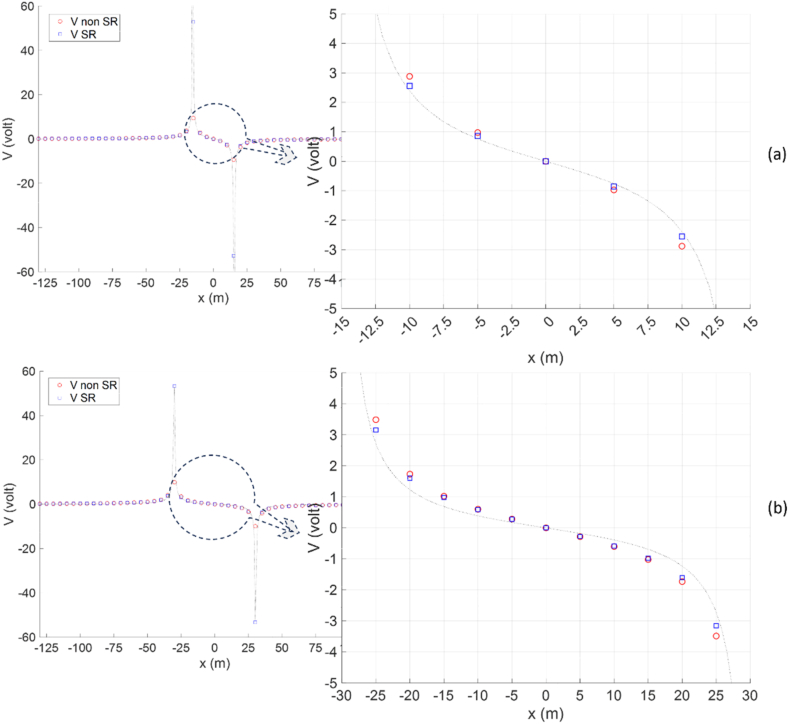
The potential curve along the *x*-axis (at *y* = 0, *z* = 0) using the Wenner array with injection current ±1A and spacing (a) *a* = 10 m, and (b) *a* = 20 m for a two-layer Earth model.

In Fig. 6a - b and Fig. 7, the Wenner array is arranged along the x-axis at *y* = 0 with the electrode array center at *x* = 0. The potential along the *x*-axis provides information about the potential distribution of each electrode, especially the potential electrodes M and N. For the Wenner and Schlumberger arrays, potential electrodes M and N are located between current electrodes A and B, so the application of the singularity removal technique significantly effects apparent resistivity. Fig. 8a - b compares potential curves along the *x*-axis for the modeling solution without the singularity removal and the modeling solution with singularity removal, represented by the Wenner array. Applying the singularity removal technique results in potential changing more slowly, but the potential rapidly increases when close to the current electrode, leading to a much higher potential near the current electrode point than without the singularity removal technique. For locations far from the current electrode (i.e., more than three nodes), the potential values between both. Consequently, for the Wenner and Schlumberger arrays with small spacing, the application of the singularity removal technique promises much better accuracy of apparent resistivity.

For modeling benchmarking purposes, an accuracy test of the application of singularity removal technique in 3D DC geoelectric resistivity modeling, based on orthosceme element, is conducted. This test focuses on the apparent resistivity of the Wenner and Schlumberger arrays in two-layer and three-layer Earth models. The apparent resistivity *ρ*_*a*_ of the Wenner array is given by Eq. (19),

(19) $$\rho_{a}=2\pi a\frac{\Delta V}{I}$$

where *a* is the electrode spacing, *I* is the injection current, and the potential value Δ*V* is obtained numerically. The analytical formulation of apparent resistivity *ρ*_*a*_ of the Wenner array for two-layer Earth model has been derived by Telford et al. [36] and further elaborated by Eluwole et al. [41],

(20) $$\rho_{a}=\rho_{1}\left[1+4\sum_{n=1}^{\infty }\frac{k^{n}}{\sqrt{1+D^{2}}}-\frac{k^{n}}{\sqrt{4+D^{2}}}\right],k=\frac{\rho_{2}-\rho_{1}}{\rho_{2}+\rho_{1}},D=\frac{2nd}{a}$$

where *ρ*_*1*_ is the resistivity of layer 1, *ρ*_*2*_ is the resistivity of layer 2, and *d* is the thickness of layer 1. For three-layer Earth model, the apparent resistivity is analytically calculated by modifying Eq. (20). The apparent resistivity is calculated step-by-step for each pair of layers to obtain the final apparent resistivity as in Eq. (21),

(21) $$\rho_{a12}=\rho_{1}\left[1+4\sum_{n=1}^{\infty }\frac{{k_{1}}^{n}}{\sqrt{1+{D_{1}}^{2}}}-\frac{{k_{1}}^{n}}{\sqrt{4+{D_{1}}^{2}}}\right],k_{1}=\frac{\rho_{2}-\rho_{1}}{\rho_{2}+\rho_{1}},D_{1}=\frac{2nd_{1}}{a}\;\rho_{a}=\rho_{3}\left[1+4\sum_{n=1}^{\infty }\frac{{k_{2}}^{n}}{\sqrt{1+{D_{2}}^{2}}}-\frac{{k_{2}}^{n}}{\sqrt{1+{D_{2}}^{2}}}\right],{\;k}_{2}=\frac{\rho_{3}-\rho_{a12}}{\rho_{3}+\rho_{a12}}{,D}_{2}=\frac{2n\left(d_{1}+d_{2}\right)}{a}$$

where *ρ*_*a12*_ is the apparent resistivity of layer 1 and layer 2, *ρ*_*a*_ is the apparent resistivity of layer 1, layer 2, and layer 3, *ρ*_*1*_ is the resistivity of layer 1, *ρ*_*2*_ is the resistivity of layer 2, *ρ*_*3*_ is the resistivity of layer 3, *d*_*1*_ is the thickness of layer 1, and *d*_*2*_ is the thickness of layer 2.

**Fig. 9:**
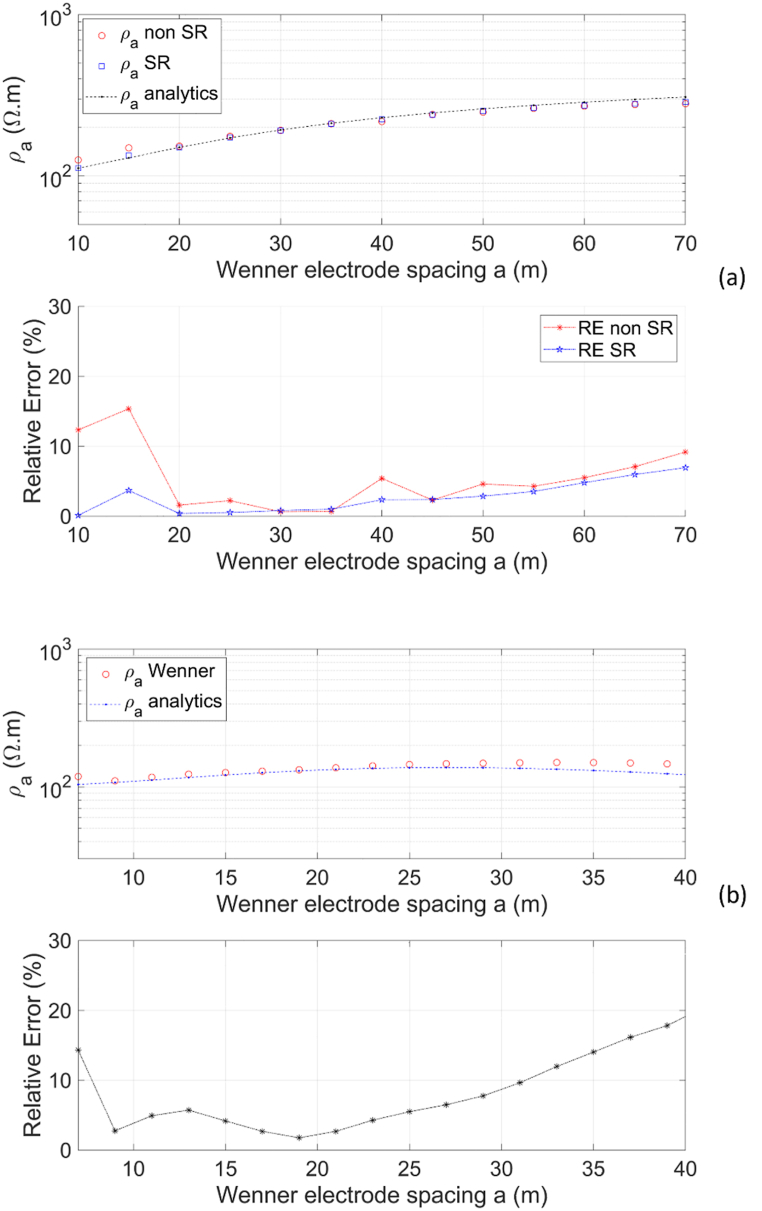
Apparent resistivity curve of the Wenner array and relative error of numerical apparent resistivity to analytical for (a) two-layer Earth model, and (b) three-layer Earth model.

Numerical modeling of 3D DC geoelectric resistivity using the Wenner array is applied to the layered Earth models (two-layer and three-layer Earth models) with spacing *a* = 10–70 m, repeated at intervals of 5 m and a fixed center point electrode array at *x* = 0, producing the apparent resistivity curve in Fig. 9a. At small spacing, the apparent resistivity is close to the resistivity of the first layer with *ρ*_*1*_ = 100 Ωm. As spacing increases, the apparent resistivity rises due to the influence of second layer with *ρ*_*2*_ = 500 Ωm. At small spacings, it can be observed that the numerical solution without singularity removal technique produces a higher apparent resistivity than the analytical solution, with a relative error of up to >10 %. This is consistent with the description in Fig. 8aandb, where the potential solution is close to the current electrode, leading to singular values. By using the singularity removal technique, the numerical solution provides apparent resistivity that matches the analytical results with a relative error value of <5 % for spacings from 10 to 60 m. The visualization in Fig. 9a is considered representative enough of the singularity removal technique's performance, so it does not need to be performed again for other models and arrays. Fig. 9b shows the numerical solution of modeling with the singularity removal technique for the three-layer Earth model, which provides apparent resistivity that varies with electrode spacing. At small spacing, the apparent resistivity is predominantly controlled by the resistivity of the first layer, i.e., *ρ*_*1*_ = 100 Ωm. As electrode spacings become larger, the apparent resistivity increases due to the influence of the resistivity of the second layer *ρ*_*2*_ but then decreases again due to the influence of the third layer *ρ*_*3*_. A similar trend of apparent resistivity for the three-layer Earth model is shown by the modeling results conducted by Jaysaval et al. [42]. The relative error <5 % only occurs at spacings of approximately 10–25 m, indicating that the analytical approach for the apparent resistivity of the Wenner array in the three-layer Earth model (Eq. (21)) needs to be re-evaluated. Gouda et al. [43] suggest that selecting the calculation step of apparent resistivity requires comparing the initial apparent resistivity $\rho_{a12}$ and the final apparent resistivity $\rho_{a}$. Another analytical approach utilizes SimPEG analytical solutions, a massively parallel modeling run on supercomputers [42], which is obviously impossible on portable computers.

Benchmarking of the 3D DC geoelectric resistivity modeling using a singularity removal technique using orthosceme element-based finite element method on layered Earth models was also conducted using the Schlumberger array. The electrode layout of the Schlumberger array follows the rules in Fig. 4b, with the electrode array center fixed but applied to layered Earth model in Fig. 6aandb. As mentioned earlier, the patterns of current flow and potential distribution closely resemble those obtained using the Wenner array. The discussion directly addresses the apparent resistivity curve. The apparent resistivity formula of the Schlumberger array is Eq. (22),

(22) $$\rho_{a}=\frac{\pi \left(L^{2}-l^{2}\right)}{2l}\frac{\Delta V}{I}$$

where *L* is half the length of the current electrode AB, *l* is half the length of the potential electrode MN, *I* is the injection current, and Δ*V* is obtained numerically. The apparent resistivity of the Schlumberger array is analytically obtained by solving the Hankel integral as in Eq. (23),

(23) $$\rho_{a}=L^{2}\int_{0}^{\infty }T\left(\lambda \right)J_{1}\left(\lambda L\right)\lambda \;d\lambda$$

where *J*_*1*_ denotes the first-order Bessel function of first kind, *T* denotes the Schlichter kernel, and *λ* denotes the integral variable [37].

**Fig. 10:**
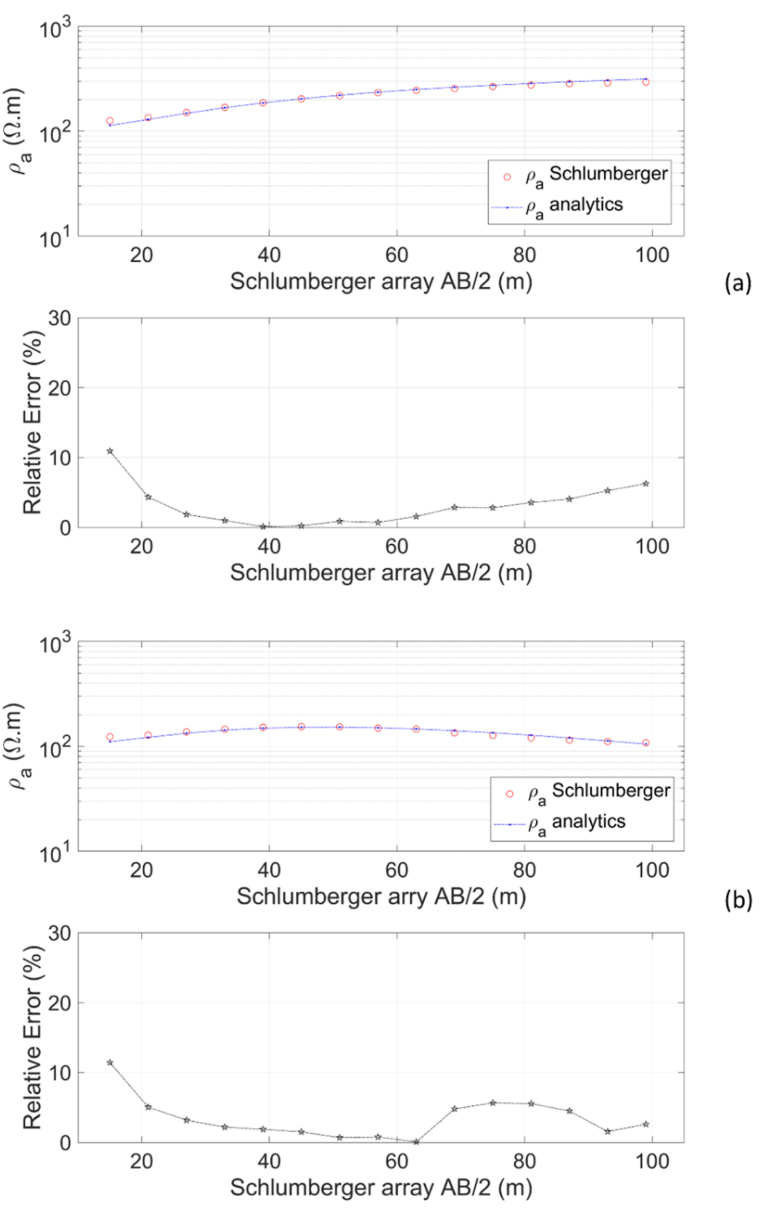
Apparent resistivity curve of the Schlumberger array and relative error of numerical apparent resistivity to analytical for (a) two-layer Earth model, and (b) three-layer Earth model.

The Schlumberger array rule is applied to a layered Earth model (two-layer and three-layer Earth models in Fig. 6aandb) with a fixed MN/2 of 3 m and AB/2 varying from 15 to 99 m with an interval of 6 m (following the formula AB/2=(2n+1)∙MN/2) and a fixed electrode array center at *x* = 0. Fig. 10 shows the apparent resistivity curves of the Schlumberger array and its relative errors for varied half spacing of current electrodes AB/2. As with the Wenner array, the apparent resistivity curve of the Schlumberger array for the two-layer Earth model (Fig. 10a) exhibits a similar pattern. At small AB/2, the apparent resistivity is close to the resistivity of the first layer *ρ*_*1*_ = 100 Ωm. The larger AB/2, higher the apparent resistivity due to the influence of the second layer resistivity *ρ*_*2*_ = 500 Ωm. For AB/2 varying from 20 to 90 m, the apparent resistivity is accurate with a relative error of <5 %. The apparent resistivity curve of the Schlumberger array for the three-layer Earth model (Fig. 10b) follows a similar trend to the Wenner array. Based on Fig. 10b, as AB/2 increases, the apparent resistivity tends to increase due to the influence of the resistivity of the second layer with *ρ*_*2*_ = 500 Ωm, then decreases due to the influence of the resistivity of the third layer with *ρ*_*3*_ = 10 Ωm. The accuracy of the Schlumberger array apparent resistivity for the three-layer Earth model is excellent, with most relative errors being <5 %, except in some AB/2 spacings.

### 6.2 Vertical contact earth model and buried block 3D anomaly earth model

**Fig. 11:**
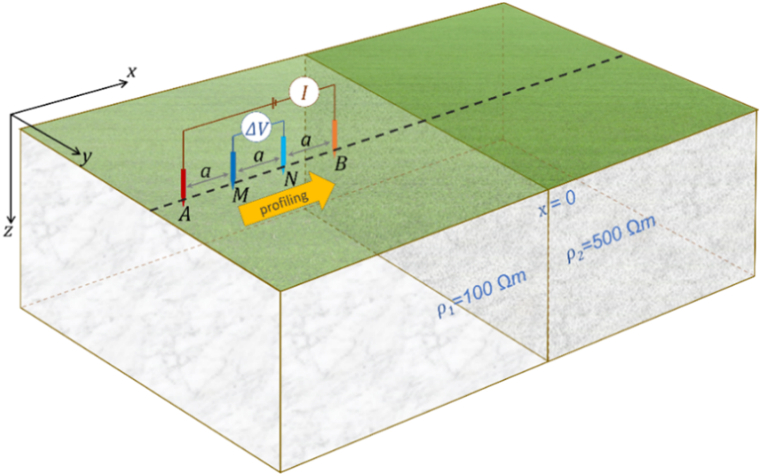
The Wenner array on a vertical contact Earth model with a profiling line along the ***x***-axis at *y* = 0 m, *x* = [-50, 50] m. The Schlumberger and dipole-dipole arrays profiling are also applied to this Earth model.

After benchmarking the 3D DC geoelectric resistivity modeling with the singularity removal technique using orthosceme element-based finite element method and obtaining sufficiently small relative errors of apparent resistivity (mostly <5 %), this modeling is further applied to a vertical contact Earth model and a buried block 3D anomaly Earth model. As shown in Fig. 11, a vertical contact Earth model features laterally varying resistivity. The boundary between medium 1 with resistivity *ρ*_1_ = 100 Ωm and medium 2 with resistivity *ρ*_2_ = 500 Ωm is a vertical plane at *x* = 0. The vertical contact Earth model is typically used to represent fault structures. In the lateral profiling implementation of the Wenner array (Fig. 11), electrode spacing is maintained at *a* = 6 m, and the electrode array center is shifted laterally from *x* = −50 to 50 m along the *x*-axis at *y* = 0. This vertical contact Earth model also applies the Schlumberger and dipole-dipole arrays profiling. The Schlumberger array rule in Fig. 4b with AB/2 = 9 m and MN/2 = 1.5 m and dipole-dipole array rule in Fig. 4c with *a* = 1 m and *n* = 9 are applied to the vertical contact Earth model with the electrode array center also shifted laterally. The application of lateral profiling with the three electrode arrays was conducted to investigate how sensitive the influence of medium resistivity is to the apparent resistivity of each electrode array.

**Fig. 12:**
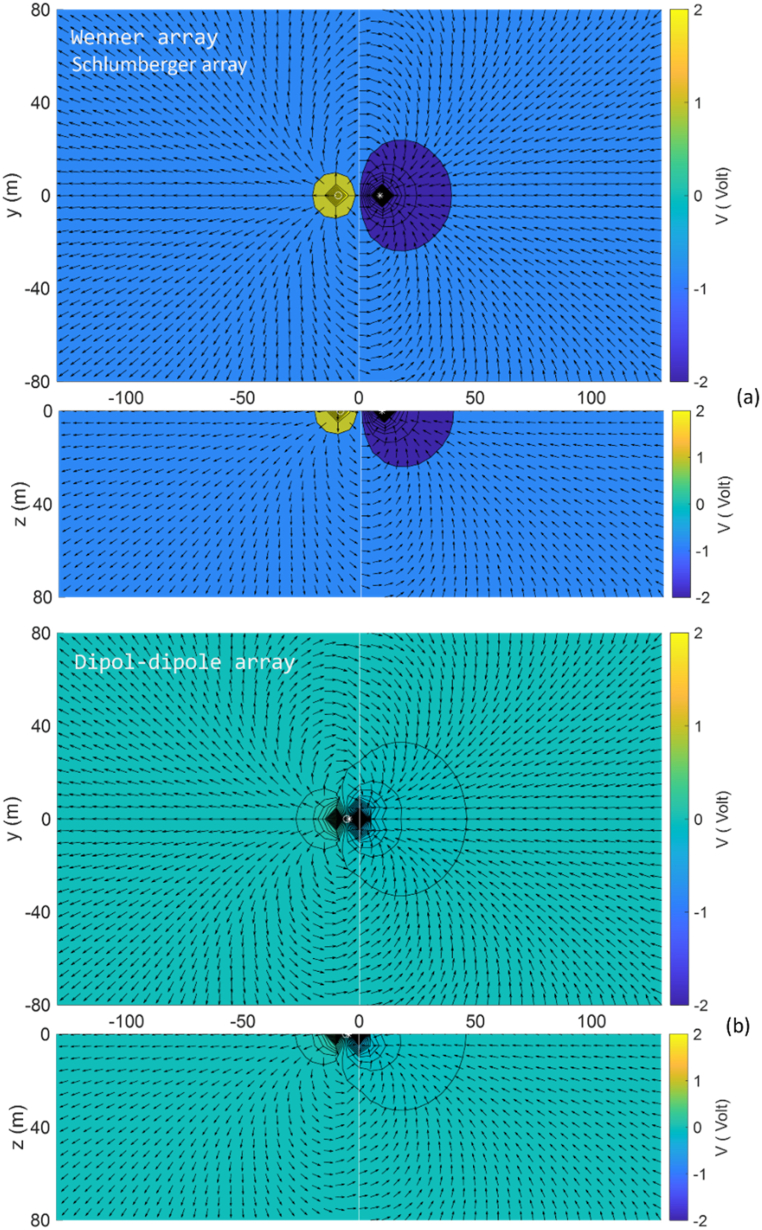
Normalized current flow and potential distribution for the vertical contact Earth model in surface view and intersection view at *y* = 0 using (a) the Wenner array, with spacing *a* = 6 m and the Schlumberger array, with AB/2 = 9 m and MN/2 = 1.5 m, and (b) the dipole-dipole array, with spacing *a* = 1 m and *n* = 9.

The solution for the 3D DC geoelectric resistivity modeling with the singularity removal technique for the vertical contact Earth model is presented as normalized current flow and potential distribution patterns in both surface view and intersection view at *y* = 0, this is illustrated along the Wenner, Schlumberger, and dipole-dipole array lines for lateral profiling at point *x* = 0 (Fig. 12). The Wenner array, with spacing *a* = 6 m, and the Schlumberger array, with AB/2 = 9 m, produce the same position of both current electrodes, resulting in similar visualizations of current flow and potential distribution patterns (Fig. 12a). In contrast, the dipole-dipole array, with spacing *a* = 1 m and n = 9, places the current electrodes closer to the left of the electrode array center, as shown in Fig. 12b, leading to quite different normalized current flow and potential distribution patterns. In the conductive medium (*ρ*_1_ = 100 Ωm), the potential distribution decreases more rapidly than in the resistive medium (*ρ*_2_ = 500 Ωm). In Fig. 12a, potential distribution pattern around the positive current electrode exhibits a faster

decay due to its location in a more conductive medium, compared to the negative electrode area. In the dipole-dipole array (Fig. 12b), changes in the direction of current flow and potential distribution patterns are observed as it passes through a medium boundary, i.e., a vertical plane with different resistivity. Changes in current flow near the vertical border between the two mediums affect their potential [44].

**Fig. 13:**
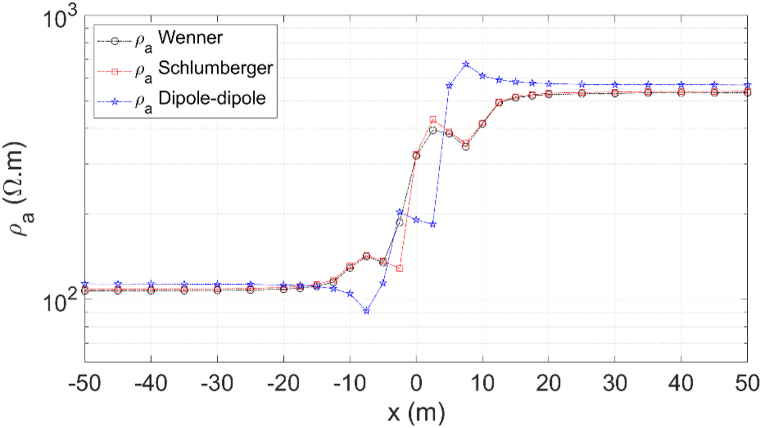
Apparent resistivity profile of the Wenner, Schlumberger, and dipole-dipole arrays for the vertical contact Earth model with the profiling line along the *x*-axis at *y* = 0 from *x* = [-50, 50] m.

As shown in Fig. 13, this lateral profiling application using the Wenner, Schlumberger, and dipole-dipole arrays along the x-axis at *y* = 0 for *x* = [−50, 50] m produces apparent resistivity profiles. The effects of potential changes due to variations in current flow when crossing the vertical boundary plane of the medium are recorded by the potential electrode, impacting on its apparent resistivity. The apparent resistivity for the three electrode arrays shows a transition from a low value near the conductive medium (*ρ*_1_ = 100 Ωm) to a significant change near the medium boundary plane, ultimately crossing into the resistive medium (*ρ*_2_ = 500 Ωm). The sensitivity of the electrode arrays to vertical structures is evident in the apparent resistivity change patterns near the medium boundary plane. Srigutomo et al. [28] utilized the Wenner array and Penz et al. [14] employed the Schlumberger array for the vertical contact Earth model, both producing similar apparent resistivity profiles to those in this study. A notably different apparent resistivity profile occurs with the dipole-dipole array. The apparent resistivity decreases to a value lower than that of the resistive medium (*ρ*_*a*_ < 100 Ωm) when approaching the medium boundary, then rapidly increases to a value higher than that of the conductive medium (*ρ*_*a*_ > 500 Ωm) upon crossing the boundary plane. The apparent resistivity profile changes in the dipole-dipole array are more contrasting than those of the other two electrode arrays. Binley and Slater [45] summarized that the dipole-dipole array is more suitable for lateral structural investigations than the other arrays.

**Fig. 14:**
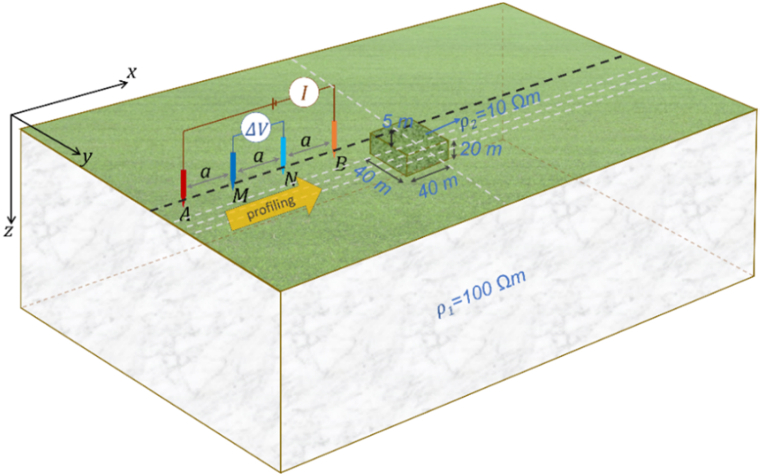
Wenner array on a buried block 3D anomaly Earth model with the profiling line along the *x*-axis at *y* = 0, ranging from *x* = −100 to 100 m. Dashed white lines indicate profiling line with spacing *a* = 15 m along the *x*-axis at *y* = 0, 15 m, 20 m, 25 m, and along the *y*-axis at *x* = 0.

The 3D DC geoelectric resistivity modeling with the singularity removal technique was also applied to a buried block 3D anomaly Earth model (Fig. 14) to provide a more realistic scenario. A 3D block with size *x* = 40 m, *y* = 40 m, *z* = 20 m, and resistivity *ρ*_2_ = 10 Ωm is placed at the center of the modeling domain, with a medium resistivity *ρ*_1_ = 100 Ωm at a depth of 5 m from the surface. The apparent resistivity profile for this Earth model was obtained using the Wenner, Schlumberger, and dipole-dipole arrays profiling. The visualization of the Wenner array profiling line, with spacing *a* = 15 m along the *x*-axis at *y* = 0, 15 m, 20 m, 25 m, and along the *y*-axis at *x* = 0, is shown in Fig. 14 as a dashed white line. This also applies to the other arrays: the Schlumberger array, with reference to Fig. 4b (where AB/2 = 18 m and MN/2 = 1.5 m), and the dipole-dipole, with reference to Fig. 4c (where *a* = 3 m and *n* = 6). The implementation of five profiling lines for the three arrays aims to illustrate the 3D apparent resistivity profile due to the buried block 3D anomaly.

**Fig. 15:**
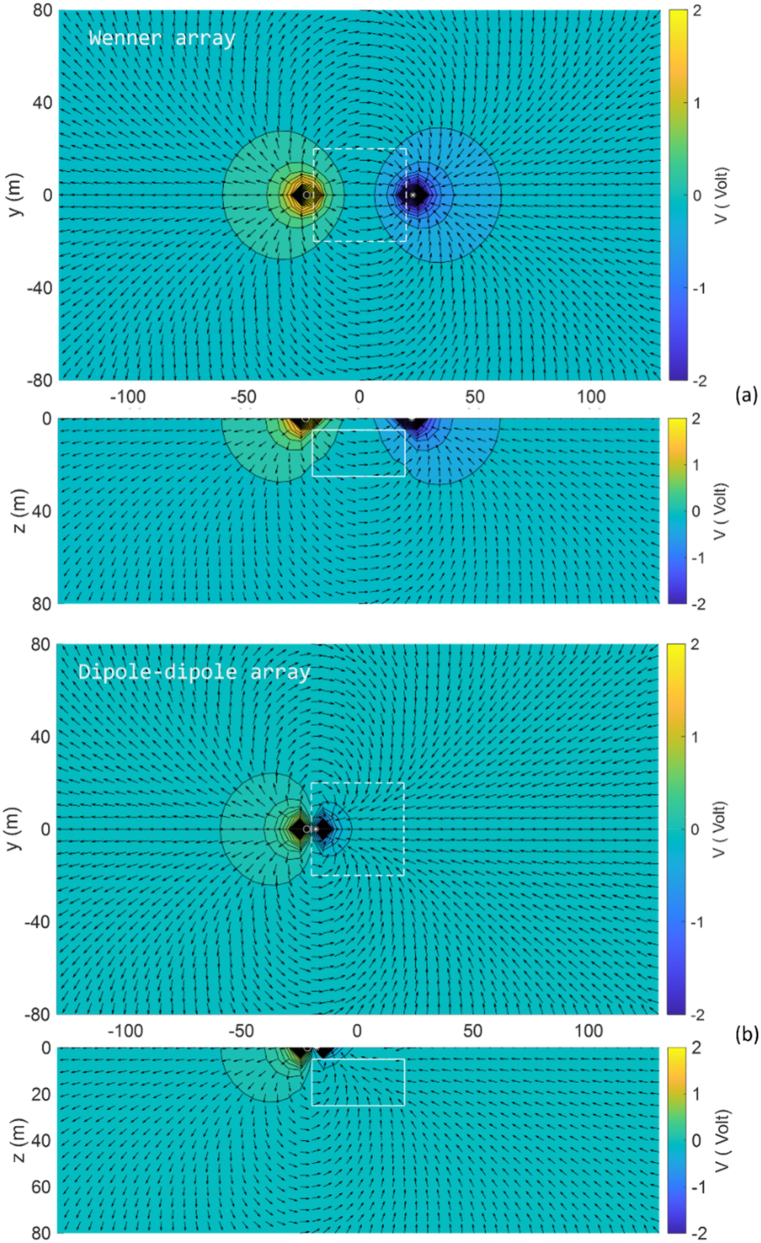
Normalized current flow and potential distribution for the 3D anomaly Earth model in surface view and intersection view at *y* = 0 using (a) the Wenner array with spacing *a* = 15 m, and (b) the dipole-dipole array with spacing *a* = 4 m and *n* = 9.

Visualization of the modeling solution for the buried block 3D anomaly Earth model as normalized current flow and potential distribution patterns is demonstrated in Fig. 15 in surface view and intersection view at *y* = 0 for lateral profiling at *x* = 0. The Wenner array, with spacing *a* = 15 m, and the Schlumberger array, with AB/2 = 18 m and MN/2 = 1.5 m, produce similar patterns of normalized current flow and potential distribution thus they are sufficiently represented by the Wenner array in Fig. 15a. In contrast, the normalized current flow and potential distribution patterns of the dipole-dipole array, with the current electrodes located close together with *a* = 4 m and *n* = 9, are presented in Fig. 15b. Generally, the current flow direction and potential distribution patterns change upon entering the 3D anomaly due to variations in current near the boundary faces of the anomaly.

**Fig. 16:**
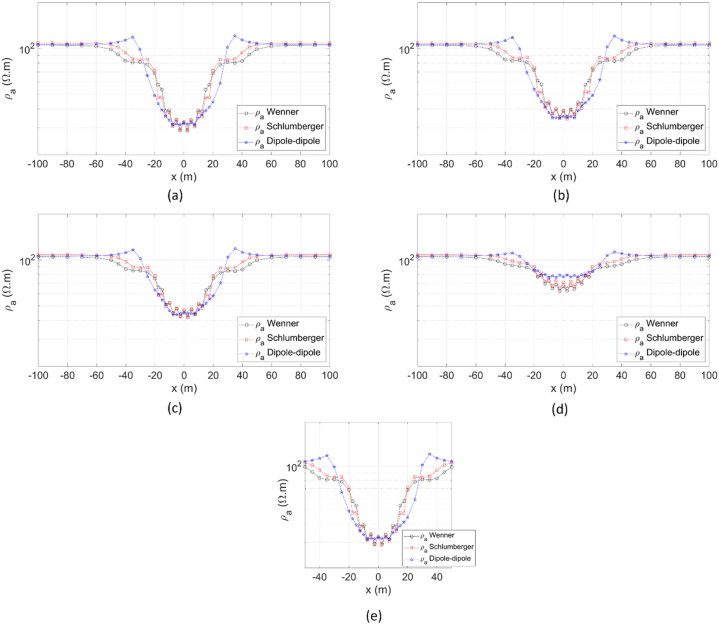
Apparent resistivity profile of the Wenner, Schlumberger, and dipole-dipole arrays for a buried block 3D anomaly Earth model with profiling lines along the *x*-axis at (a) *y* = 0, (b) *y* = 15 m, (c) *y* = 20 m, (d) *y* = 25 m from *x* = [-100, 100] m, and (e) along *y*-axis at *x* = 0 from *y* = [-50, 50] m.

Lateral profiling using the Wenner, Schlumberger, and dipole-dipole arrays was conducted to evaluate the performance of the 3D DC geoelectric resistivity modeling with singularity removal technique on a buried block 3D anomaly Earth model. Schaa et al. [15] previously demonstrated that the apparent resistivity of the dipole-dipole array can effectively to represent the presence of a more conductive buried block 3D anomaly, indicated by a decrease in apparent resistivity when it intersects the anomaly. Based on the evaluation in Fig. 16a–e, the apparent resistivity profiles for the three electrode arrays began to respond to the presence of the buried block 3D anomaly starting at 40 m before and after the anomaly, showing a decrease in apparent resistivity. In Fig. 16a and e, where profiling lines are perpendicular to each other and pass through the center of the 3D anomaly, the apparent resistivity decreases to approximately 30 Ωm due to the influence of the more conductive buried block. As the profiling line approaches the boundary faces of the buried block anomaly, the apparent resistivity continues to decrease. The apparent resistivity profile along the *x*-axis at *y* = 25 m in Fig. 16d indicates that the influence of the buried block anomaly is still present, with a decrease in apparent resistivity 60 to 70 Ωm, even though the profiling line is outside the 3D anomaly. The change in apparent resistivity profile patterns for the Wenner array is nearly identical to that of the Schlumberger array, in contrast to the dipole-dipole array, particularly at boundary faces of the buried block anomaly. Overall, the apparent resistivity profiles of the three electrode arrays effectively represent the presence of buried block anomaly.

## 7 Conclusion

The 3D DC geoelectric resistivity modeling with the singularity removal technique is performed by numerically solving the secondary potential equation using an orthosceme based finite element method, while the primary potential is solved analytically. Utilizing the PCG solver with incomplete Cholesky factorization (ICF) as a preconditioner to manage the global matrix for a current electrode position enhances the computational speed by up to 6.7 times compared to the direct solver, while maintaining accuracy. This improvement is particularly significant for specific electrode arrays with numerous current electrode positions. The 3D visualization of the modeling solution illustrates the trend of potential changes with varying distances and medium conductivities. The potential curve along the sounding line for both the Wenner and Schlumberger arrays confirms that the singularity removal techniques effectively address the singularity in potential values around the current electrodes, as evidenced by the rapid rise in the potential value when approaching the current electrodes. These potential values are further utilized to derive the apparent resistivity of the selected electrode array. Benchmarking this modeling on a layered Earth model using the apparent resistivity of the Wenner and Schlumberger arrays indicates that the 3D DC resistivity geoelectric modeling with the singularity removal technique achieves a relative error of <5 % for the Wenner array spacings of 10–60 m. The Schlumberger array performs even better, with nearly all relative errors remaining <5 %. In a vertical contact Earth model, the apparent resistivity profiles of both the Wenner and Schlumberger arrays exhibit similar patterns, with resistivity increasing toward the vertical boundary plane and approaching the resistivity of the second medium. In contrast, the dipole-dipole array shows more pronounced changes near the vertical boundary, making it more suitable for detecting lateral structures. The buried block 3D anomaly Earth model provides a realistic scenario for this study, with the apparent resistivity profiles of the Wenner, Schlumberger, and dipole-dipole arrays effectively representing the presence of the 3D anomaly.

## CRediT authorship contribution statement

**Supriyadi:** Writing – original draft, Visualization, Software, Methodology, Conceptualization. **I.G.P.F. Soerya Djaja:** Validation, Software. **Elfitra Desifatma:** Visualization, Methodology. **Harry Mahardika:** Writing – review & editing, Methodology, Conceptualization. **Wahyu Srigutomo:** Writing – review & editing, Validation, Supervision, Conceptualization.

## Data availability statement

The data related to this study have not been deposited in any publicly accessible repository. However, the datasets generated in this study are available upon request to the corresponding author.

## Funding

This research was funded by Indonesian Education Scholarships (BPI) provided by The Center for Higher Education Fund (BPPT) or Center of Education Services (Pusat Layanan Pendidikan) 10.13039/501100023174Ministry of Education, Culture, Research, and Technology of the Republic of Indonesia and the Indonesia Endowment Fund for Education (LPDP) under contract no. 0848/J5.2.3/BPI.06/10/2021 awarded to the first author.

## Declaration of competing interest

The authors declare that they have no known competing financial interests or personal relationships that could have appeared to influence the work reported in this paper.

## Figures and Tables

**Table 2:** Direct solver vs. PCG with ICF as a preconditioner performance for different numbers of nodes and elements.

Number of nodes (*x* × *y* × *z*)	Number of elements	Direct solver time (s)	ICF generation time (s)	PCG + ICF generation		
				Error	Iteration	Time(s)
49 × 33 × 16	138240	0.248654	0.001419	9 × 10^−7^	37	0.034887
59 × 39 × 20	251256	0.366394	0.002381	7.7 × 10^−7^	43	0.054628
71 × 45 × 22	388080	0.621886	0.003744	9.2 × 10^−7^	47	0.092073

## Appendix A Performance of PCG with Modified Incomplete Cholesky Factorization (ICF) as a Preconditioner

The performance of the direct solver and the PCG with modified ICF as a preconditioner is evaluated using the global matrix from Eq. (16) at various sizes. The difference in the sizes of the global matrix is achieved by changing the number of nodes (and elements). The variations in the number of nodes used are 49 × 33 × 16, 59 × 39 × 20 (used in this study), and 71 × 45 × 22, all within a fixed modeling domain of *x* = [−300, 300] m, *y* = [−250, 250] m, and *z* = [0, 250] m, which uses a layered Earth model as Fig. 6a. The time recorded in each column of Table 2 represents the average runtime in seconds over twenty runs. The “PCG + ICF generation” time (last column in Table 2) refers to the total time for both the ICF generation and the solving step of Eq. (16). The data in Table 2 exhibits that for all variations of the number of nodes, the PCG with ICF can solve Eq. (16) at least 6.7 times faster than the direct solver, with an error tolerance of 10^−7^.
